# Commentary: Virtual *In-Silico* Modeling Guided Catheter Ablation Predicts Effective Linear Ablation Lesion Set for Longstanding Persistent Atrial Fibrillation: Multicenter Prospective Randomized Study

**DOI:** 10.3389/fphys.2017.01113

**Published:** 2017-12-22

**Authors:** Axel Loewe, Olaf Dössel

**Affiliations:** Institute of Biomedical Engineering, Karlsruhe Institute of Technology (KIT), Karlsruhe, Germany

**Keywords:** atrial fibrillation, catheter ablation, virtual ablation, computational model, *in-silico* modeling

Shim et al. (2017) presented a prospective study on ablation therapy guided by computational modeling results in the current “Frontiers in Physiology” research topic “Clinical Application of Physiome Models.” In their study, patients with persistent atrial fibrillation (AF) scheduled for ablation therapy were randomized to have either the physician or a computational model determine the best ablation strategy out of a predefined set of five strategies. They derived patient-specific anatomical surface models from left atrial CT images and induced atrial fibrillation (AF) in the model by applying a specific rapid pacing protocol. A human operator applied point-wise virtual ablation to the models by clicking in a graphical user interface according to each of the five strategies: circumferential pulmonary vein isolation (CPVI), CPVI + posterior box ablation ± anterior line, CPVI + roof line + left lateral isthmus line, CPVI + CFAE ablation based on the electrograms derived from the model. The ablation strategy that led to earliest termination of AF in the simulation was deemed optimal and applied in the patients.

Prospectively guiding catheter ablation for AF by means of personalized computational models is a long desired (Krueger et al., 2013b; Boyle et al., 2016; Jacquemet, 2016), giant leap in the field of computational cardiology that we want to congratulate on. Succeeding to do so in a multicenter study in particular is a great accomplishment in terms of logistical, procedural, and presumably, traditionalistic challenges. Shim et al. report a duration of <6 h from the time the CT was available to the time the model suggested the optimal ablation strategy and all manual work was performed during core working hours. Their results show that it is feasible to leverage computational modeling in a clinical time scale and under clinical constraints. Besides feasibility, Shim et al. proved that computationally guided ablation is not inferior in terms of procedure duration and complication rate. These are great findings that will hopefully fuel translational efforts to exploit computational models in the field of cardiac electrophysiology (Boyle et al., 2017). Despite these promising results, we want to highlight that the results regarding efficacy of the ablation therapy (comparable recurrence rates as for empirical ablation) should be considered specific for the rather simplistic approach employed to choose a particular ablation pattern using the computational model.

The models used by Shim et al. were individualized only in terms of the anatomy of the endocardial wall. Differences in myocardial wall thickness have been reported to influence AF dynamics (Biktasheva et al., 2015; Whitaker et al., 2016). These, as well as potential dissociation between layers in the atrial wall (Verheule et al., 2014) can per definition not be considered in a surface model of excitation spread. Moreover, the properties of the atrial substrate play a crucial role for virtually all mechanisms discussed to be potentially involved in AF perpetuation. According to the circus movement reentry concept (Schotten et al., 2011), the wavelength defined as the product of the conduction velocity (CV) and the duration of the effective refractory period (ERP) plays a crucial role. The wavelength in relation to the atrial surface determines to a large share how many reentrant activities can be sustained on a given anatomical model (Deng et al., 2017). Thus, reduction of wavelength (due to CV or ERP decrease) could compensate for a large share of the effects of an enlarged LA (Qu, 2006). Intracardiac electrograms can be used to derive patient-specific information regarding the CV (Weber et al., 2011; Cantwell et al., 2015), the ERP (Corrado et al., 2017), zones of slow conduction or block (Trächtler et al., 2015), and low voltage areas as a surrogate for fibrotic regions (Jadidi et al., 2016). However, time constraints cause the spatial resolution of these measurements to be rather coarse and they can only be derived during the procedure. Thus, either the time window for the computational evaluation would shrink significantly or the measurements would need to be acquired during an extra procedure. Substrate information that can be obtained non-invasively is late gadolinium-enhanced MRI as a surrogate for fibrotic tissue. Particularly the spatial distribution of fibrotic tissue has been shown to crucially impact AF dynamics in computational models (McDowell et al., 2015; Zahid et al., 2016a). Other aspects that have been shown to affect AF dynamics and can be considered in models as population-level a-priori knowledge is gross myocyte orientation (Wachter et al., 2015) and regional electrophysiological heterogeneity (Colman et al., 2013; Krueger et al., 2013a). A non-homogeneous atrial substrate is a prerequisite when simulating signals to compute meaningful CFAE maps (Ashihara et al., 2012; Keller et al., 2013). Known gene mutations (Loewe et al., 2014) and e.g., blood electrolyte levels (Krueger et al., 2011) give room for further personalization.

In addition to the possible improvements regarding model fidelity and personalization, the virtual ablation method and assessment of its success give opportunities for further optimization. The set of ablation strategies to choose from was rather limited in the study by Shim et al. and other approaches could be required to obtain optimal results (Bayer et al., 2016; Zahid et al., 2016b). Probably even more important, termination of the specific AF episode that was induced in the model as a sole success criterion might not be sufficient to predict long-term success. Other aspects to consider are reinducability of (potentially different) AF episodes and vulnerability to subsequently develop atrial flutter, which is a common clinical complication.

In conclusion, we would like to point out that the work by Shim et al. is an outstanding example of translating computational modeling to the clinical environment and encourage everyone to follow down this road. On the other hand, we want to highlight that the results on efficacy of computationally guided ablation should be considered a lower bound rather than a representative example of what value personalized electrophysiological modeling can potentially add. We hope that the work by Shim et al. will fuel the development and facilitate the use of more sophisticated models under clinical constraints to leverage the full power of computational modeling approaches in the near future.

## Author contributions

All authors listed have made a substantial, direct and intellectual contribution to the work, and approved it for publication.

### Conflict of interest statement

The authors declare that the research was conducted in the absence of any commercial or financial relationships that could be construed as a potential conflict of interest.
